# Novel Strategy with Gemcitabine for Advanced Pancreatic Cancer

**DOI:** 10.5402/2011/936893

**Published:** 2011-05-23

**Authors:** Shuji Komori, Shinji Osada, Kazuhiro Yoshida

**Affiliations:** ^1^Department of Surgical Oncology, Gifu University Graduate School of Medicine, Yanagido, 1-1 Yanagido, Gifu 501-1194, Japan; ^2^Department of Surgery, Ibi Welfare Hospital, Ibi, Gifu 501-0619, Japan

## Abstract

5-fluorouracil (5-FU) is widely used in chemotherapy for gastric and colorectal cancer, but gemcitabine (GEM), and not 5-FU, is approved as a standard drug for use in pancreatic cancer. Interindividual variation in the enzyme activity of the GEM metabolic pathway can affect the extent of GEM metabolism and the efficacy of GEM chemotherapy. Human equilibrative nucleoside transporter 1 (hENT1) is recognized as a major transporter of GEM into cells. In addition, a factor that activates hENT1 is the inhibition of thymidylate synthase (TS), one of the 5-FU metabolic enzymes; TS inhibition mediates depleting intracellular nucleotide pools, resulting in the activation of the salvage pathway mediated through hENT1. In this paper, the role of 5-FU in GEM-based chemotherapy for pancreatic cancer is discussed with special emphasis on enzymes involved in the 5-FU and GEM metabolic pathways and in the correlation between GEM responsiveness and the expression of 5-FU and GEM metabolic enzymes.

## 1. Introduction

Pancreatic cancer is one of the most life-threatening cancers; 35,240 deaths in Americans in 2009 (6% of all United States cancer deaths) make this cancer one of the leading causes of cancer-related death [1]. In spite of recent progress in surgical procedures, the operative resectability rate of pancreatic cancer remains unsatisfactory at 9% to 20% [2, 3]. Development of chemotherapeutic modalities has shifted from 5-fluorouracil (5-FU), one of the primary standard drugs used to treat solid cancers, to gemcitabine (GEM, Gemzar; Eli Lilly and Company, Indianapolis, Ind), the most anticipated agent for the treatment of this problematic disease [4]. However, treatment results and favorable outcomes with GEM remain variable; the response rate with GEM ranges from 5.4% to 16.7% [5, 6], and the median survival time (MST) of patients treated with 5FU of 4.2–4.5 months [5] is extended by GEM to 5.9–6.5 months [4, 6]. The concept of single-agent chemotherapy is clearly limited, and novel approaches to combination therapy should be considered. Here, we describe our recent challenges in pancreatic cancer and review the chemotherapeutic procedures currently available for the treatment of pancreatic cancer. 

## 2. Application of Biological Study to Pancreatic Cancer Therapy

Recently, the study of the cell signaling pathway has been applied to the control of cancer proliferation, invasion, and metastasis as a molecular targeted therapy [7]. Among them, vitamin K3 (menadione), which induces cell apoptosis through activation of oxidative stress, has also been expected as a unique anticancer drug for pancreatic cancer [8, 9]. In the process of bringing experimental studies of such agents to the clinical trial stage, a drug delivery system is currently under consideration [10]. In parallel with the development of these future biochemical trials, recent studies based on standard chemotherapy with GEM, have also been developed.

Nucleoside transporters are commonly known to include two equilibrative nucleoside transporters (ENT1/2) and three concentrative nucleoside transporters (CNT1/2/3) [11]. Recent kinetic studies of human cell lines have shown the intercellular uptake of GEM to depend mainly on ENT1, which localizes in plasma and mitochondrial membranes [11–13]. ENT1 activity was reported to be a prerequisite for the occurrence of the growth inhibitory effect of GEM, because cells deficient in ENT1 activity were highly resistant to GEM; the rate of growth inhibition was increased 39- to 1800-fold in the presence of ENT1 [12]. Expression of mRNA and proteins was also evaluated as a favorable predictor of the effect of GEM clinically; MST was longer in patients with high versus low expression, 25.7 versus 8.5 months with mRNA expression (*P* = .001), and 13.0 versus 4.0 months with protein expression (*P* = .01) [14, 15]. These results suggest the action of ENT1 to be critical for GEM metabolism.

Intracellular enzymes deoxycytidine kinase (dCK), ribonucleotide reductase (RR), and 5′-nucleotidase (5′-NT) are also reported to be important in the conversion of GEM to its inactive form [16]. Some studies emphasizing the first step in limiting GEM phosphorylation by overexpression of dCK in tumor cells deficient in the enzyme have shown restoration of the response to GEM [17, 18]. RR is essential for DNA polymerization/repair [19] and consists of large and small dimerized subunits, M1 and M2, respectively. The M1 subunit possesses a binding site for enzyme regulation (regulatory subunit), and the M2 subunit is involved with RR activity (catalytic subunit) [20, 21]. Because 5′-NT reduces phosphorylated metabolites of GEM, the activity level of 5′-NT might also be a target for evaluation as the one factor most affecting the clinical outcome of GEM chemotherapy [22].

Although RRM1, dCK, and 5′-NT are useful predictors of GEM resistance [23], the individual actions of each of RR, dCK, and 5′-NT have not been reported as useful predictors of prognosis in pancreatic cancer chemotherapy. Namely, pancreatic cancer cells with a higher ratio of hENT1×dCK/RRM1×RRM2 showed higher cytotoxicity, and those cells with a lower ratio showed lower cytotoxicity [21]. Further studies are necessary to confirm the usefulness of these three factors as predictors of prognosis in pancreatic cancer.

## 3. The Role of One 5-FU Metabolic Enzyme, Thymidylate Synthase

Thymidylate synthase (TS) is generally known to be important in 5-FU metabolism [24, 25]. In solid-type carcinomas, TS expression was estimated for its ability to predict sensitivity to 5-FU; increase in the expression of 5-FU mRNA/protein resulted in resistance to 5-FU in colorectal cancer [26, 27]. Low expression of TS, as evaluated by immunohistochemistry and reverse-transcription PCR (RT-PCR), correlated with a favorable response to 5-FU-based therapy in colorectal cancer patients [25, 28, 29]. In pancreatic cancer, however, survival rate was better in the patients with high TS expression [30]. In contrast, expression of TS mRNA was found to correlate with patient survival; survival was longer in patients with low expression, but in patients with high TS expression, 5-FU-based chemotherapy showed favorable results [31]. Taken together, TS expression is related to 5-FU metabolism and its chemotherapeutic effect. In addition, Rauchwerger et al. previously reported that 5-FU itself plays a role in the inhibition of TS, and TS inhibitor modulates hENT1 [32]. On this basis, we investigated methods to improve pancreatic cancer therapy.

## 4. Challenges to the Better Treatment of Pancreatic Cancer

**Figure 1:**
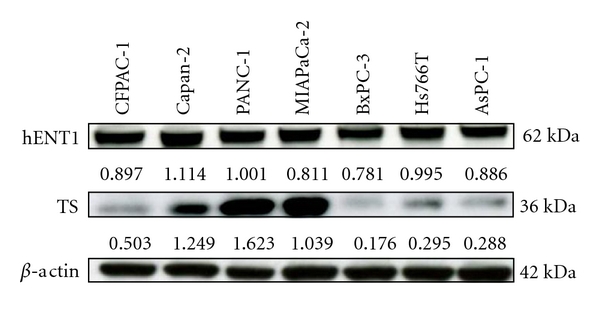
Cellular factor expression in several pancreatic cancer cell lines. Cellular factor-related metabolism for 5-FU or GEM was evaluated by Western blotting. Each value under the blotting band was obtained from comparison with the level of mouse monoclonal anti-beta-actin. hENT1, human equilibrative nucleoside transporter 1; TS, thymidylate synthase.

**Figure 2:**
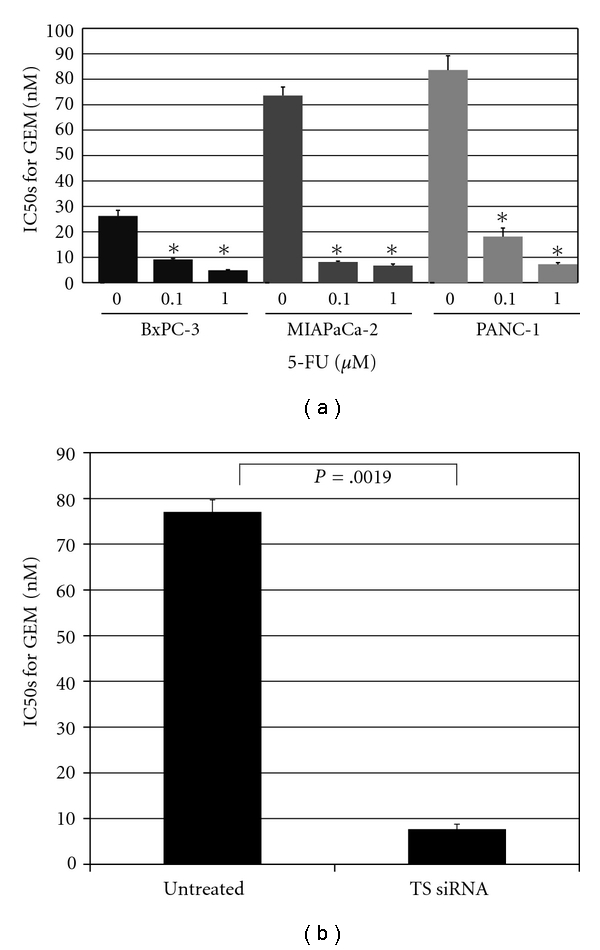
(a) Drug resistance of pancreatic cancer cells to 5-FU+GEM. Cultured cells (5 × 10^3^ cells/well) were exposed to graded concentrations of gemcitabine (0, 10^−2^–10^4^ nM) together with several 5-FU concentrations (0, 0.1, 1.0 *μ*M) for 72 h. Drug resistance was expressed as the concentration of drug that inhibited colony formation by 50% (IC50). The control for each cell line (5-FU, 0 *μ*M) showed statistical significance with each of the same cell lines (∗) (*P* < .01). (b) Effect of TS expression on GEM resistance. Knockout of TS expression significantly decreased resistance to GEM in pancreatic cancer cells as evidenced by the detected change in IC50s from 77.0 ± 2.6 nM to 7.7 ± 1.1 nM. Results are expressed as mean ± SD. Bar, SD; TS, thymidylate synthase.

**Figure 3:**
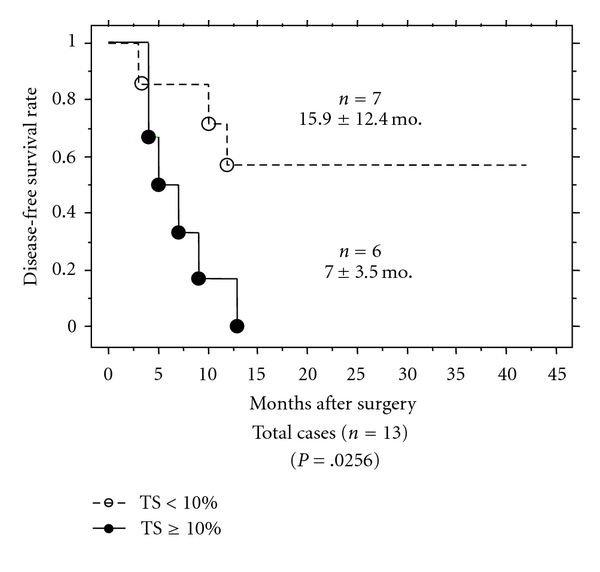
Comparison of patient disease-free survival rates. In patients receiving GEM treatment after surgery (*n* = 13), disease-free survival was compared according to percent of thymidylate synthase (TS) protein expression. Months after surgery; mean ± SD.

Expressed levels of 5-FU and/or GEM-related metabolic protein in seven independent pancreatic cancer cell lines (PANC-1, MIAPaCa-2, BxPC-3, Hs766T, Capan-2, AsPC-1, and CFPAC-1) were compared with the half maximal inhibitory concentrations (IC50s) of GEM or 5-FU, and only TS expression was found to correlate positively with drug-induced inhibitory effect on cell growth (*P* = .0169) [33]. hENT1 expression was found to be similar for each of these pancreatic cancer cell lines, whereas TS expression was found to be high in PANC-1, and moderate in MIAPaCa-2 and low in BxPC-3, as shown in (Figure 1). The relation between TS expression and GEM resistance was demonstrated by using these three pancreatic cancer cell lines (Figure 2). The inhibition of TS expression due to 5-FU (0.1 or 1.0 *μ*M, doses having no cell-inhibitory or cell-death effects) was also shown to decrease GEM resistance in a dose-dependent manner. The inhibition of TS due to siRNA on PANC-1, which is the cell line most tolerant to GEM and which shows the highest expression of TS protein, also significantly decreased the resistance of PANC-1 to GEM, showing a decrease in IC50 from 77.0 ± 2.6 nM to 7.7 ± 1.1 nM (*P* = .0019). In addition, in our clinical study of patients treated with GEM after surgery for pancreatic cancer, low expression of TS protein evaluated in resected specimens by immunohistochemical techniques was found to correlate significantly with prolongation of disease-free survival (15.9 ± 12.4 versus 7.0 ± 3.5 months, *P* = .0256), as shown in (Figure 3).

From our experience with pancreatic cancer cell lines, IC50s of 5-FU varied quite widely (9.0–1805.1 *μ*M), but the IC50s of GEM were found to be stable (6.1–77.6 nM); therefore, GEM has been accepted as a first-line chemotherapeutic drug for the treatment of pancreatic cancer [4]. In contrast, according to these studies, 5FU could be useful not only to inhibit cell growth by its chemotherapeutic actions but also to reduce TS expression to improve sensitivity to GEM.

## 5. The Benefit of Combination Treatment of Gem with 5-FU

For GEM-induced intracellular changes, is hENT1 truly the most important factor? Although a previous report indicated that hENT1 is a useful predictor of GEM responsiveness or MST of pancreatic cancer patients treated with GEM, augmentation of hENT1 mRNA was detected after GEM treatment in the cells, whereas DNA synthesis inhibitor was found to increase the activity of some nucleoside transporters at the cell surface [34]. Resistance to GEM in pancreatic cancer cell lines does not appear to involve hENT1; inhibition of hENT1-mediated transport in pancreatic cell lines modifies GEM responsiveness either modestly or not at all [34]. In addition, we have previously reported on the contribution of TS to GEM chemotherapy [33]. Indeed, as shown in (Figure 1), the differences detected in the protein expression levels of TS (0.176–1.623), but not those of hENT1 (0.781–1.114), correlated with GEM responsiveness, suggesting that hENT1 itself has no close correlation to GEM resistance. 

**Figure 4:**
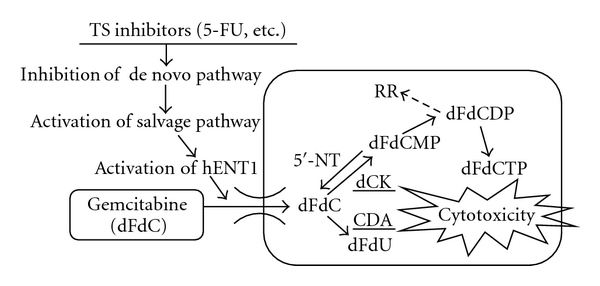
Contribution of thymidylate synthase (TS) in metabolic pathway of gemcitabine. For explanation of symbols and metabolic routes, see text. hENT1, human equilibrative nucleoside transporter; dCK, deoxycytidine kinase; RR, ribonucleotide reductase; 5′-NT, 5′-nucleotidase; CDA, cytidine deaminase.

In the metabolic pathway of GEM, not only GEM metabolic enzymes but also TS has been shown to have a direct or indirect correlation with GEM metabolism: a TS inhibitor, such as 5-FU, in the de novo pathway mediates depleting intracellular nucleotide pools, resulting in activation of the salvage pathway and hENT1 as well [13, 35] (Figure 4). The process of activating hENT1 mediates the diffusion of nucleosides, including those associated with GEM, across plasma membranes in accordance with the concentration gradient [32]. Then, because hENT1 represents as a main GEM transporter, lower expression of TS might be related to transportation of GEM due to hENT1 activation. According to our experiments, TS inhibition itself is quite critical in GEM-mediated cancer cell death and also might be useful in GEM-resistant pancreatic cancer. Decreasing protein expression of TS with 5-FU probably induces a better prognosis for the patient undergoing GEM chemotherapy.

## 6. Future Prospects

Although insufficient antitumor effect in pancreatic cancer has been detected with 5-FU alone, its modulating action in GEM treatment will be focused on in the future. In fact, the mid-trial report of the GEMSAP trial (Phase II) for Stage IV pancreatic cancer (Nakai et al.) in Japan revealed that chemotherapy with GEM plus S-1 (oral 5-FU prodrug, tegafur, gimeracil, and oteracil potassium) might be superior to chemotherapy with GEM alone (median progression-free survival, 5.4 versus 3.6 months, hazard ratio = 0.64 (95% confidence interval 0.42–0.97), *P* = .036; overall survival, 14.1 versus 8.7 months, *P* = .104) [36]. These experimental clinical results were shown by our recent study and suggested that TS and the dihydropyrimidine dehydrogenase (DPD) inhibitory effect of S-1 might produce additional effect on GEM [36]. In the future, the study of application timing and dosing of 5-FU, and S-1, that induce the most favorable outcome in regard to hENT1 should be continued. The challenge to provide better treatment against advanced pancreatic cancer is just beginning.

##  Financial Disclosure

There is no financial disclosure.
